# Impact of Different Estimation Methods on Obesity-Attributable Mortality Levels and Trends: The Case of The Netherlands

**DOI:** 10.3390/ijerph15102146

**Published:** 2018-09-29

**Authors:** Nikoletta Vidra, Maarten J. Bijlsma, Fanny Janssen

**Affiliations:** 1 Population Research Centre, Faculty of Spatial Sciences, University of Groningen, PO Box 800, 9700 AV Groningen, The Netherlands; f.janssen@rug.nl; 2 Max Planck Institute for Demographic Research, Konrad-Zuse Str. 1, 18057 Rostock, Germany; bijlsma@demogr.mpg.de; 3 Netherlands Interdisciplinary Demographic Institute, P.O. Box 11650, 2502 AR The Hague, The Netherlands

**Keywords:** obesity, mortality, The Netherlands, estimation, CRA approach, partially adjusted method, weighted sum method, population-attributable fraction

## Abstract

The available methodologies to estimate the obesity-attributable mortality fraction (OAMF) affect the levels found and hamper the construction of time series. Our aim was to assess the impact of using different techniques to estimate the levels and the trends in obesity-attributable mortality for The Netherlands between 1981 to 2013. Using Body Mass Index (BMI), all-cause and cause-specific mortality data, and worldwide and European relative risks (RRs), we estimated OAMFs using three all-cause approaches (partially adjusted, weighted sum, and the two combined) and one cause-of-death approach (Comparative Risk Assessment; CRA). We adjusted the CRA approach to purely capture obesity (BMI ≥ 30 kg/m^2^). The different approaches led to a range of estimates. The weighted sum method using worldwide RRs generated the lowest (0.9%) while the adjusted CRA approach using 2013 RRs generated the highest estimate (1.5%). Using European-specific RRs instead of worldwide RRs resulted in higher estimates. Most of the approaches revealed an increasing OAMF over the period 1981 to 2013 especially from 1993 onwards except for the adjusted CRA approach among women. Estimates of OAMF levels and trends differed depending on the method applied. Given the limited available data, we recommend using the weighted-sum method to compare obesity-attributable mortality across European countries over time.

## 1. Introduction

Over the past three decades, the prevalence of obesity has risen tremendously across the globe [1] to the point that it is now considered a pandemic [1,2]. Obesity constitutes a major health burden [3] since there is evidence of strong links between obesity and life-threatening chronic diseases such as type II diabetes, cardiovascular disease, stroke, and multiple types of cancer [4,5,6]. As a consequence, the rise in obesity has led to recent declines in survival and life expectancy [7,8]. Because the health burden associated with obesity is so significant, its estimation bears high relevance and importance.

In quantifying the health burden of obesity at the population level, the population attributable fraction (PAF) is commonly used [2]. The PAF is defined as the proportion of total events (e.g., deaths) in a population that could be prevented if a particular risk factor (e.g., obesity) could be eliminated [2]. The PAF combines information on the proportion of the population exposed to obesity (prevalence) with the relative risk (RR) of dying from obesity [9]. 

Over the years, many methodologies for estimating obesity-attributable mortality fractions (OAMF) by means of different PAF formulas have been developed and range from approaches that use RRs for all-cause mortality (all-cause approach) to more recent approaches that use RRs for obesity-related causes of death (cause-of-death approach) (See Supplementary Material file 1). Within the all-cause approach, there are various methods for estimating OAMF that require varying degrees of data availability (see the Supplementary Material file 1). Thus, implementing some of these methods can be difficult. The partially adjusted method [10,11,12,13], which multiplies the adjusted RR of dying from obesity with the obesity prevalence in the studied population, is often used [14,15]. In the weighted sum method, unadjusted RRs by age and sex (for instance) are commonly weighted by the obesity prevalence within each subgroup [16]. The Comparative Risk Assessment (CRA) methodology, which was recently developed by the Global Burden of Disease (GBD) Study, uses cause-specific mortality, cause-specific RRs, and the population distribution of BMI to estimate cause-specific shares of mortality due to a high BMI (≥23 kg/m^2^ [17]. Due to their focus on high BMI, the CRA estimates cannot be readily compared with other estimates that focus strictly on obesity (BMI ≥ 30 kg/m^2^).

As previously published research has shown, estimates of obesity-attributable mortality vary depending on which methodology is used [18]. For example, in 1991, the number of obesity-related deaths in the United States was ~196,000 when the weighted sum method was used and was ~230,000 obesity-related deaths when the partially adjusted method was applied [15]. 

The use of different methods and the range of outcomes these methods generate not only cause uncertainty about the true population-level effects of obesity on mortality in a single calendar year but also hamper the construction of time series. First, the use of different methods over time makes it difficult to construct time series of PAFs based on existing studies. Second, data limitations can also pose challenges when estimating time series. In particular, more advanced PAF methods require data that simply are not consistently available over a longer time period (see Supplementary Material file1). To date, only one previous study has examined the long-term trends in obesity-attributable mortality and did so for Canada using an all-cause approach [19]. In addition, the GBD study estimates mortality due to high BMI every five years from 1990 to 2015 [20]. Because the GBD study is updated regularly based on the latest research findings, it is unclear whether the same methodology was applied and the same cause-of-death and RR data were used in each update [20,21]. 

Previous research on obesity-attributable mortality has focused on the United States [11,13,15] in part because of the availability of large cohort studies for the US as a whole. For Europe, by contrast, there is little available information on obesity-attributable mortality levels and even less information on trends. To the best of our knowledge, the influence of the chosen method on estimates of obesity-attributable mortality trends has not previously been assessed.

Our objective is to assess the impact of the use of different estimation techniques on both the levels of and the trends in obesity-attributable mortality. More specifically, we compare approaches that can actually be used to estimate the long-term trends given the data that are available for the European context: namely, the partially adjusted method, the weighted sum method, a combined version of these methods, and the CRA approach. To enable this comparison, we adapted the CRA approach so that it calculates the PAF related to obesity only.

## 2. Materials and Methods

### 2.1. Methods for Calculating Obesity-Attributable Mortality

Below we present the different PAF formulas that we will use to estimate the obesity-attributable mortality fraction (OAMF).

### 2.2. Selected All-Cause Approaches

We follow the terminology of a partially adjusted and weighted sum method, as described by Flegal [15]. The partially adjusted method and the weighted sum method [15,16] are both all-cause approaches (see Supplementary Material file 1) that use the same PAF equation.

(1) $$\;\mathrm{PAF}=\frac{P\cdot \left(\mathrm{RR}-1\right)}{1+P\cdot \left(\mathrm{RR}-1\right)}\;$$

where P is the proportion of the population exposed to obesity and RR is the (unadjusted) relative risk of mortality associated with obesity [14]. The partially adjusted method combines one overall adjusted RR of dying from obesity with the observed overall obesity prevalence. The weighted sum method uses unadjusted subgroup-specific RRs and subgroup-specific prevalence.

In fact, the weighted sum method commonly uses a modified version of Equation (1), which distinguishes multiple categories of the exposure variable such as different age groups.

(2) $$\;\mathrm{PAF}=\frac{\sum P_{i}\cdot \left({\mathrm{RR}}_{i}-1\right)}{1+\;\sum P_{i}\cdot \left({\mathrm{RR}}_{i}-1\right)}$$

where *i* refers to the *i*th exposure category.

In addition to the above-mentioned approaches, we also used a combined all-cause approach in which we used age-specific and sex-specific obesity prevalence and age-adjusted and sex-specific RRs in Equation (2). A single PAF value is achieved through weighting by sex.

### 2.3. Comparative Risk Assessment (CRA) Approach

The Comparative Risk Assessment Approach (CRA) (terminology following Flegal [17]) estimates the number of cause-specific deaths that would be prevented if the current BMI distributions were changed to a hypothetical alternative distribution: the so-called counterfactual distribution [22]. We rewrote Equation (2) to show more intuitively how this approach calculates mortality attributable to high BMI (≥23 kg/m^2^).

(3) $$\;\mathrm{PAF}=\frac{\sum p_{1i}{\mathrm{RR}}_{i}-\sum p_{2i}{\mathrm{RR}}_{i}}{\sum p_{1i}{\mathrm{RR}}_{i}}\;$$

where $p_{1}$ refers to the observed BMI distribution (the factual distribution), $p_{2}$ to the counterfactual distribution, and ${\mathrm{RR}}_{i}$ to the cause-specific relative risk of mortality for the exposure level *i*. 

### 2.4. Adjustment of the CRA Approach

To estimate deaths attributed to obesity only (BMI ≥ 30 kg/m^2^), we adjusted the CRA calculation to the following equation.

(4) $$\;{\mathrm{PAF}}_{\mathrm{BMI}\geq 30\;}=\;\frac{\sum_{\mathrm{BMI}\geq 30}p_{1i}\cdot {\mathrm{RR}}_{i}-\sum_{\mathrm{BMI}\geq 30}p_{2i}\cdot {\mathrm{RR}}_{i}}{\sum_{i=0}^{\infty }p_{1i}\cdot \mathrm{RR}\;}$$

i.e., we limited the numerator to categories with BMI 30+ while the denominator contains all BMI categories so that the resulting fraction can be multiplied by the total deaths in a country. As counterfactual, we used the BMI range between 21 and 23 kg/m^2^ [20,21]. 

### 2.5. Data Sources

#### 2.5.1. BMI Data

Obesity prevalence and BMI distribution (i.e., Mean ± Standard Deviation) data required for the all-cause and the CRA approaches, respectively, were obtained from the Dutch Health Interview Survey [23] from Statistics Netherlands. This is a nationally representative on-going study based on self-reported weight and height covering the period between 1981 and 2013 [23,24]. 

#### 2.5.2. Mortality Data

All-cause mortality data by sex, age group (30–75+), and year (1981 to 2013) were obtained from the Human Mortality Database (HMD) [25] and were used for all the approaches. 

For the adjusted CRA approach, we obtained cause-specific mortality data by sex and age from the World Health Organization (WHO) Mortality Database [26]. In fact, we used two different sets of death causes and associated RRs (see Table 1) and—consequently—distinguish between two adjusted CRA approaches: adjusted CRA recent and adjusted CRA less recent. See Table 1 for the causes of death used in these two adjusted CRA approaches.

### 2.6. Relative Risks (RRs)

Since unadjusted RRs are not readily available, we had to include adjusted RRs for the all-cause approaches, which has been done previously [10,11]. From the meta-analysis by Flegal et al., we obtained worldwide and European overall RRs—adjusted for (at least) age and sex—and sex-specific RRs [27]. From the meta-analysis by Wang, we obtained age-specific and sex-specific worldwide RRs [28]. From the Dynamo project [29], which is based on a comprehensive literature review, we obtained European age-specific and sex-specific RRs (see Table 2).

## 3. Results

The different approaches generated different estimates of the OAMFs for The Netherlands in 2013 (see Table 3). Using worldwide RRs, the weighted sum method provided the lowest estimates for men and women combined (0.92%) and for men (0.86%). However, the partially adjusted method provided a slightly lower estimate for women (0.94%) than the weighted sum method (0.98%). The adjusted CRA approach using the 2013 world RRs generated the highest estimates for men and women combined (1.46%) and for women (1.62%) while the combined all-cause method using the world RRs provided the highest estimate for men (1.43%). The use of European-specific RRs instead of worldwide RRs resulted in higher estimates. The weighted sum method using the European-specific RRs resulted in the highest estimates overall (1.78%). 

**Figure 1:**
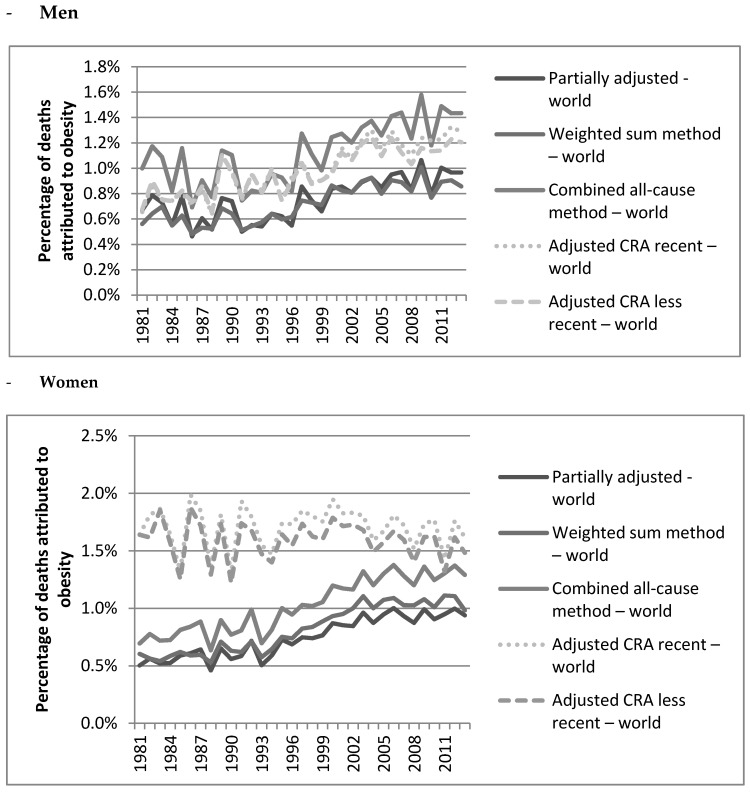
Estimates of the percentage of deaths attributed to obesity in The Netherlands using worldwide RRs from 1981 to 2013.

Overall, the different approaches—with the exception of the results for women generated by the CRA approaches—showed that the OAMF levels increased over the period 1981 to 2013 and especially from 1993 onwards (see Figure 1, Table 4). For men, the trends are parallel for the different approaches even though, in terms of the percentage change, the CRA approaches resulted in larger overall increases (>75%) (Table 4) than the all-cause approaches (around 50%). For women, the trends identified by the adjusted CRA approaches clearly differed from those found by the other approaches. Over the period 1981 to 2013, both the adjusted CRA approach with recent RRs and the adjusted CRA with less recent RRs resulted in a decline (−1.7% and −9.6%, respectively) as well as with a slight increase from 1993 onwards (4.3% and 0.7%, respectively). For women, the other approaches estimated the overall percentage increase at around 85% even though the partially adjusted method using European RRs resulted in a larger increase (133%) and the weighted sum method using worldwide RRs in a smaller increase (63%). When applied to men and women combined (Figure S1), the partially adjusted method and the weighted sum method produced very similar levels and trends from 1993 onwards. The same was observed for the two CRA approaches.

## 4. Discussion

### 4.1. Summary of Results

The different approaches to estimate obesity-attributable mortality fractions (OAMFs) for The Netherlands in 2013 led to a range of estimates. The weighted sum method using worldwide RRs generated the lowest values (0.9%) while the adjusted CRA approach using 2013 RRs generated the highest estimate (1.5%). Using European-specific RRs instead of worldwide RRs resulted in higher estimates. Most of the approaches revealed an increasing OAMF over the period 1981 to 2013 especially from 1993 onwards. However, the adjusted CRA approach showed that there was hardly any increase among women. 

### 4.2. Explanation of the Observed Results

The different approaches we applied—i.e., the partially adjusted method, the weighted sum method, the combined all-cause method, and the adjusted CRA approach—provided different estimates of obesity-attributable mortality. These findings further corroborate previous research showing that PAF estimates vary widely, according to the methodology used [18]. The plausible explanations for our finding that the estimates produced by the CRA approaches were higher than the estimates generated by the partially adjusted method and the weighted sum method lie not only in the methodology employed but also in the different underlying data and RRs used. Specifically, in the all-cause approaches, obesity prevalence, all-cause mortality data, and all-cause RRs [15] are used while, in the adjusted CRA approach, more detailed information is used including data on the whole BMI distribution, cause-specific mortality, and the cause-specific RRs [30]. It appears that the combined all-cause method was able to produce similar results based on less detailed information.

Our finding for 2013 that the weighted sum method produced lower OAMF estimates when worldwide RRs are used seems to be in line with the results of a study conducted in the US in 1991, which showed that the partially adjusted method generated an OAMF estimate that was 17% higher than the estimate produced by the weighted sum method [15]. The explanation given by the researchers is that the partially adjusted method does not fully account for confounding and effect modification by age and sex. It should be noted, however, that, in our analysis, the RRs for the partially adjusted method and the weighted sum method did not come from the same study. This most likely explains why the partially adjusted method actually produced lower estimates than the weighted sum method when the European RRs were used. 

In our study, the use of European RRs resulted in higher estimates than the use of worldwide RRs within the same method primarily because the European RR values were higher than the worldwide RR values (see Table 2). Additionally, in previous literature, European RR values were reported to be higher than those from North America, East Asia, Australia, and New Zealand [10,20,27]. While no clear explanation of these differences in RR values has been provided in the available literature, it most likely follows the notion that RRs could be different for some disease and all-cause mortality across geographical contexts [29,31]. In other words, the differences in RR values across populations are likely related to variation in dietary [32], disease, and mortality patterns and in the genetic background [33]. 

In our study, all of the approaches (except of the adjusted CRA approach for women) revealed an increase in obesity-attributable mortality levels especially from 1993 onwards. These findings are in line with the results of previous studies of obesity prevalence and BMI mean values for The Netherlands, which showed that obesity prevalence increased sharply after the 1990s (see Figure S2) [24]. In addition, the accelerated increase is in line with the observations for other European adult cohort populations [34]. Taken together, these findings seem to indicate that the obesity epidemic is accelerating in The Netherlands and other European populations. It should be noted, however, that the GBD study found that the trend in mortality due to high BMI in The Netherlands was decreasing over the 1990–2013 period. This seemingly contradictory finding is most likely related to the indirect estimation by the GBD of mean BMI values over time. The declining trend that was found using their indirect estimation [35] does not reflect the actual observed trend in The Netherlands [24]. 

For women, the adjusted CRA approach clearly resulted in different trends compared to the other approaches with a decline over the period 1990 to 2013. Since these differences were not observed for men, the reason for this finding seems to lie less in the methodology that was applied than in the sex-specific data that were used. Since the RRs do not change over time and are fairly similar for men and women (see Table 1), the trends in mean BMI and the trends in cause-specific mortality are more decisive. Specifically, we found that the mean BMI values of women have increased less than the mean BMI values of men in all age groups (see Figure S3). Moreover, when we looked at the trends for ischemic and cerebrovascular heart disease, which represent around 70% of the changes in mortality in both sexes, we found that, after 1993 ischemic and cerebrovascular mortality declined more among women than among men. 

### 4.3. Reflection on Our Approach

When aiming to compare the different methodologies to estimate obesity-attributable mortality and their estimates concerning Europe, data availability is a major restricting factor. Related to this, we chose a practical and rather straightforward evaluation strategy where we compared the methodologies that can practically be applied with the available data and their point estimates.

Each available method requires a different level of detail with regard to the RRs (by age group or not, by cause of death or not). Unfortunately, a dataset providing all the RRs needed is not available. Instead, we, like previous studies, had to rely on RRs from different data sources/meta-analyses for each method [18], which is likely to have an impact on our results. The comparison of levels is likely to generate different outcomes (different differences) when, in a simulation study, hypothetical RRs from one source are used. The comparison of trends will, however, likely be much less affected because distorting factors presumably lead to larger RR differences between sources than within sources over time. More importantly, however, when the method is applied in practice, it will do so based on the available RRs. 

In addition, we chose not to estimate confidence intervals surrounding the RRs. First, due to using secondary data (from different sources, and both for RR and prevalence), we do not possess the covariance matrix of the relevant variables. Hence, a formal comparison of levels or trends cannot be made unless untestable assumptions—which will affect the comparisons—are also made [18]. Second, in standard frequentist approaches, confidence intervals cannot capture all the uncertainty surrounding the estimation of obesity-attributable mortality. For instance, they cannot assess the uncertainty regarding the choice of the RR. Thirdly, policy makers, public health professionals, and researchers working on obesity-attributable mortality in Europe mostly use point estimates. 

Although we used a fairly simple evaluation approach, it is still the first time that the influence of the chosen method (and the related RRs) on obesity-attributable mortality trends for a European country has been assessed. In doing so, our study provided valuable insights into the pros and cons of the different methods and highlighted as well the urgency for additional data in Europe.

### 4.4. Reflection on the Different Methodologies

We applied methodologies that, based on their data requirements, enabled us to construct time series of the OAMF. We included three all-cause approaches: the partially adjusted method, the weighted sum method, and a combined all-cause method. The partially adjusted method requires the least amount of detailed data. However, because of the scarcity of unadjusted RRs, most studies that have applied this method (including our study) used adjusted RRs instead [10,11], which can produce biased estimates [15]. To limit this bias, we used the combined all-cause method, which enabled us to weigh by sex. We also applied the weighted sum method, which allowed us to take into account both confounding and effect modification by age and sex using age-specific and sex-specific RRs. As the weighted sum method deals with confounding and effect modification better than the other all-cause methods we examined, it seems to be the most appropriate method to use for the estimation of OAMF. In fact, the results produced by the weighted sum method using European RRs were close to the results generated in 2013 by the adjusted CRA method.

In addition, we applied the CRA approach, which we adjusted to estimate the share of mortality due to obesity only. An advantage of the CRA approach is that it has the potential to provide more reliable estimates because it takes more information into account such as information on the obesity-related causes of death. One disadvantage of the CRA approach is that it requires the BMI distribution and data on (detailed) causes of death, which are not readily available for European countries. This issue is especially salient when aiming to study time trends. In addition, the changes in the ICD classification over time [36] make it difficult to obtain the complete list of causes of death associated with obesity over time. 

Given these considerations, we recommend the use of the weighted sum method for the study of trends in OAMFs for the European context and ideally the provision of confidence intervals. 

## 5. Conclusions 

Estimates of both the levels of, and the trends in, obesity-attributable mortality fractions in The Netherlands differed depending on the method applied as well as on the underlying data and the relative risks (RRs) used. Since obesity prevalence is relatively low in The Netherlands, we would expect to find even larger differences for countries with higher obesity prevalence. In quantifying the health burden of obesity at the population level, it is, therefore, essential to compare different methodologies and different RRs. 

Comparisons of obesity-attributable mortality between countries and over time can only be performed accurately by using the same methodology as well as comparable data and RRs. Since the data that are currently available for Europe are limited, we recommend using the weighted-sum method and European RRs to compare obesity-attributable mortality across European countries and over time. 

## Figures and Tables

**Table 1:** Causes of death used in the two adjusted CRA approaches and the associated RRs ranging from ages 50 to 54.

Adjusted CRA Less Recent			Adjusted CRA Recent		
Danaei et al. 2009 *	Relative Risks		GBD 2013 **	Relative Risks	
Causes of Death	Men Aged 50–54	Women Aged 50–54	Causes of Death	Men Aged 50–54	Women Aged 50–54
Colon and rectum cancers	1.04	1.02	Colon and rectum cancers	1.03	1.01
Breast cancer	-	1.02	Breast cancer	-	1.02
Corpus uteri cancer	-	1.10	Corpus uteri cancer	-	1.10
Diabetes mellitus	1.20	1.20	Diabetes mellitus	1.21	1.21
Hypertensive heart disease	1.18	1.18	Hypertensive heart disease	1.18	1.18
Ischemic heart disease	1.09	1.09	Ischemic heart disease	1.09	1.09
Cerebrovascular disease	1.10	1.10	Cerebrovascular disease	1.10	1.10
Kidney cancer	1.05	1.05	Kidney cancer	1.04	1.06
Pancreatic cancer	1.01	1.02	Pancreatic cancer	1.01	1.02
			Esophageal cancer	1.07	1.06
			Liver cancer	1.05	1.03
			Gallbladder cancer	1.03	1.06
			Leukemia	1.02	1.02

* The causes of death we included are the same as those listed by Danaei 2009 [22] except that we did not include non-Hodgkin lymphoma. ** The GBD 2013 also uses the following causes of death: hemorrhagic stroke, cardiomyopathy, atrial fibrillation, aortic aneurysm, peripheral vascular endocarditis, other cardiovascular disease, diabetes, chronic kidney disease (CKD), glomerulonephritis CKD, other CKD, hypertensive CKD, ovarian cancer, and thyroid cancer [21]. However, because our study period covers causes of death classified by both ICD-9 and ICD-10 and some of the abovementioned detailed causes of death were not available from the WHO mortality database, we restricted ourselves to the causes of death listed here.

**Table 2:** RRs used in the all-cause approach and their characteristics.

Approach	Geographical Context	Age	RR		Reference
			Men	Women	
Partially adjusted	Worldwide	All adult ages	1.18		Flegal et al. 2013 [27]
Partially adjusted	Europe	All adult ages	1.27		Flegal et al. 2013 [27]
Combined approach	Worldwide	All adult ages	1.27	1.25	Flegal et al. 2013 [27]
Weighted sum method	Europe	<50	1.55	1.5	Lobstein & Leach 2010 [29]
		50–59	1.539	1.49	
		60–69	1.5225	1.475	
		70+	1.495	1.45	
Weighted sum method	Worldwide	< 35	1.59	1.60	Wang 2015 [28]
		35–44	1.39	1.58	
		45–54	1.39	1.49	
		55–64	1.21	1.35	
		65–74	1.15	1.25	
		75+	1.11	1.11	

For our adjusted CRA approach, we used cause-specific, sex-specific, and age-specific RRs provided by the GBD 2013, which are worldwide RRs based on a meta-analysis [21] (recent RRs). In addition, we identified previously published worldwide RRs based on a meta-analysis [22] (less recent RRs) (see Table 1).

**Table 3:** Estimates of the percentage of deaths attributed to obesity by method and sex in The Netherlands in 2013.

Approach	Men	Women	Men and Women
Partially adjusted – world	0.97	0.94	1.00
Partially adjusted – Europe	1.45	1.37	1.41
Weighted sum method – world	0.86	0.98	0.92
Weighted sum method – Europe	1.88	1.68	1.78
Combined all-cause method – world	1.43	1.29	1.37
Adjusted CRA, recent – world	1.29	1.62	1.46
Adjusted CRA, less recent – world	1.21	1.48	1.35

**Table 4:** Percentage change in obesity-attributable mortality fractions (OAMF) from 1981–1993, 1993–2013, and 1981–2013 in The Netherlands by sex.

Change in OAMF (%)	1981–1993	1993–2013	1981–2013
Men			
Partially adjusted - world	−19.1%	78.7%	44.5%
Partially adjusted – Europe	−14.8%	73.2%	47.5%
Weighted sum method – world	2.3%	49.3%	52.7%
Weighted sum method – Europe	−13.6%	74.4%	50.6%
Combined all-cause method – world	−19.2%	77.8%	43.6%
Adjusted CRA recent – world	25.3%	56.4%%	96.0%
Adjusted CRA less recent – world	19.5%	48.9%	77.9%
**Women**			
Partially adjusted - world	0.26%	85.9%	86.4%
Partially adjusted – Europe	31.5%	77.1%	133.0%
Weighted sum method – world	−4.8%	70.8%	62.6%
Weighted sum method – Europe	2.1%	79.5%	83.3%
Combined all-cause method – world	0.2%	85.2%	85.6%
Adjusted CRA recent – world	−5.8%	4.3%	−1.7%
Adjusted CRA less recent – world	−10.3%	0.7%	−9.6%

## Supplementary Materials

The following are available online at http://www.mdpi.com/1660-4601/15/10/2146/s1, Materials file 1: Literature review of available methods for calculating obesity-attributable mortality by means of the Population Attributable Fraction (PAF), Material file 2: Supplementary Figures and Tables. Figure S1: Estimates of the percentage of male and female deaths combined attributed to obesity in The Netherlands, using world RRs, 1981–2013, Figure S2: Age-standardised obesity prevalence by sex, 20–75+ yrs. in The Netherlands, 1981–2013, Figure S3: BMI Mean values, 30–79 years, The Netherlands, 1981–2013, Table S1: PAF estimates compared in men and women for The Netherlands 1981, 1991, 2001 and 2013, and 1981–2013.
