# Supplementary material for: Impact of Different Estimation Methods on Obesity-Attributable Mortality Levels and Trends: The Case of The Netherlands
Source: Int J Environ Res Public Health. 2018 Sep 29;15(10):2146. doi: 10.3390/ijerph15102146 (PMC6210009; doi:10.3390/ijerph15102146)
Supplement: Supplementary file 1 [file ijerph-15-02146-s001.zip › Supplementary material 1 final.docx]

**Impact of Different Estimation Methods on Obesity-Attributable Mortality Levels and Trends: The Case of the Netherlands**

**Nikoletta Vidra ^1,*^, Maarten J. Bijlsma ^2^, Fanny Janssen ^1,3^**

1. **Population Research Centre, Faculty of Spatial Sciences, University of Groningen, PO Box 800, 9700 AV, Groningen, The Netherlands.**
2. **Max Planck Institute for Demographic Research, Konrad-Zuse str. 1, 18057, Rostock, Germany.**
3. **Netherlands Interdisciplinary Demographic Institute, The Hague, The Netherlands P.O. Box 11650, 2502 AR, The Hague.**

*** Corresponding author: E-mail:** [**n.vidra@rug.nl**](mailto:n.vidra@rug.nl)

**Supplementary Material 1: Literature review of available methods for calculating obesity-attributable mortality by means of the Population Attributable Fraction (PAF)**

To identify the available methods for calculating obesity-attributable mortality by means of the Population Attributable Fraction (PAF), we searched the US National Library of Medicine Gateway system, PubMed Central, and PubMed to identify the relevant published literature using the following search terms: obesity or BMI or body-mass-index and attributable or PAF or attributable and mortality. After reviewing the available literature, we identified five different PAF formulas for estimating obesity-attributable mortality; each formula requiring different data (see Table 1). We divided the methods into those that use relative risks (RRs) for all-cause mortality (all-cause approach) and those that use RRs for obesity-related causes of death (cause-of-death approach).

**Table 1: Methods to estimate the obesity-attributable mortality fraction (OAMF) by means of different PAF formulas and their data requirements**

| Type of method | Formula used to calculate PAF | Required data |
| --- | --- | --- |
| All-cause approach, Allison DB et al. 1999 and Banegas JR 2003 [1, 2 ] | $PAF=\frac{P\cdot\left( RR-1 \right)}{1+P\cdot\left( RR-1 \right)}$ | Proportion of population exposed to obesity (P);  Relative risk (RR) of mortality associated with obesity, not adjusted for confounding. |
| Cause-of-death approach, Farzadfar F et al. 2011 [*3*] | $PAF=\frac{\sum p_{1i}\mathrm{RR}_{i}-\sum p_{2i}\mathrm{RR}_{i}}{\sum p_{1i}\mathrm{RR}_{i}}$ | Observed BMI distribution (Mean ± Standard Deviation) ($p_{1i}$);  Counterfactual BMI distribution ($p_{2i}$)†;  Cause-specific relative risks (RR) of mortality corresponding to obesity level i. |
| All-cause approach, Flegal KM 2005[4] | $PAF=\frac{M-M^{*}}{M}$  where  $M=I\Sigma p_{i}\mathrm{RR}_{i}$  $M^{*}=I\Sigma p_{i}\mathrm{RR}_{i}*$ | Population baseline mortality rate (I)  Relative risks (RR_i_) corresponding to each combination of BMI level and the levels of the accounted covariates for each age-group (i);  Counterfactual relative risk (RR_i_*)† when BMI is set to the reference level but other risk factors are left unchanged;  Prevalence of BMI-risk factor combinations (p_i_) |
| All-cause approach, Katzmarzyk PT et al. 2004[5] | $PAF=P_{d} \cdot\frac{(RR-1)}{\mathrm{RR}}$ | Proportion of the deceased exposed to obesity (P_d_);  Relative risk (RR) of mortality associated with obesity, adjusted for relevant confounders |
| All-cause approach, Masters RK et al. 2013[6] | $\mathrm{PAF}_{\mathrm{ik}}=[\mathrm{pd}_{\mathrm{ik}} \cdot(\mathrm{HRoverweight}_{k}-1)/ \mathrm{HRoverweight}_{k})]+[\mathrm{pd}_{\mathrm{ij}} \cdot((HRgrade1_{k}-1)/HRgrade1_{k})]+[ \mathrm{pd}_{\mathrm{ik}} \cdot((HRgrade{23}_{k}-1)/HRg{23}_{k})]$ | Fraction of total deaths that are exposed to the ith 5-year birth cohort at age k (pd_ik_)  Hazard ratio of overweight mortality risk to normal weight mortality risk at age k (HRoverweight_k_)  Hazard ratio of grade 1 obesity mortality risk to normal weight mortality risk at age k (HRgrade1_k_)  Hazard ratio of grade 2/3 obesity mortality risk to normal weight mortality risk at age k (HRgrade23_k_). |

† If the counterfactual BMI distribution or the counterfactual relative risk is theoretical, instead of the distribution or relative risk of e.g. a comparison group, it is not strictly speaking an empirical data requirement.

References

1. Allison, D. B.; Fontaine, K. R.; Manson, J. E.; Stevens, J.; VanItallie, T. B. Annual deaths attributable to obesity in the United States. *JAMA* **1999***, 282*, 1530-1538.

2. Banegas, J. R.; Lopez-Garcia, E.; Gutierrez-Fisac, J. L.; Guallar-Castillon, P.; Rodriguez-Artalejo, F. A simple estimate of mortality attributable to excess weight in the European Union. *Eur. J. Clin. Nutr.* **2003***, 57*, 201-208.

3. Farzadfar, F.; Danaei, G.; Namdaritabar, H.; Rajaratnam, J. K.; Marcus, J. R.; Khosravi, A.; Alikhani, S.; Murray, C. J.; Ezzati, M. National and subnational mortality effects of metabolic risk factors and smoking in Iran: a comparative risk assessment. *Popul. Health. Metr* **2011***, 9*, 55-7954-9-55.

4. Flegal, K. M.; Graubard, B. I.; Williamson, D. F.; Gail, M. H. Excess deaths associated with underweight, overweight, and obesity. *JAMA* **2005***, 293*, 1861-1867.

5. Katzmarzyk, P. T.; Ardern, C. I. Overweight and obesity mortality trends in Canada, 1985-2000. *Can. J. Public Health* **2004***, 95*, 16-20.

6. Masters, R. K.; Reither, E. N.; Powers, D. A.; Yang, Y. C.; Burger, A. E.; Link, B. G. The impact of obesity on US mortality levels: the importance of age and cohort factors in population estimates. *Am. J. Public Health* **2013***, 103*, 1895-1901.
