# Supplementary material for: Impact of Different Estimation Methods on Obesity-Attributable Mortality Levels and Trends: The Case of The Netherlands
Source: Int J Environ Res Public Health. 2018 Sep 29;15(10):2146. doi: 10.3390/ijerph15102146 (PMC6210009; doi:10.3390/ijerph15102146)
Supplement: Supplementary file 1 [file ijerph-15-02146-s001.zip › Supplementary material 2 final.docx]

**Impact of Different Estimation Methods on Obesity-Attributable Mortality Levels and Trends: The Case of the Netherlands**

**Nikoletta Vidra ^1,*^, Maarten J. Bijlsma ^2^, Fanny Janssen ^1,3^**

1. **Population Research Centre, Faculty of Spatial Sciences, University of Groningen, PO Box 800, 9700 AV, Groningen, The Netherlands.**
2. **Max Planck Institute for Demographic Research, Konrad-Zuse str. 1, 18057, Rostock, Germany.**
3. **Netherlands Interdisciplinary Demographic Institute, The Hague, The Netherlands P.O. Box 11650, 2502 AR, The Hague.**

*** Corresponding author: E-mail:** [**n.vidra@rug.nl**](mailto:n.vidra@rug.nl)

**Supplementary Material 22 – Supplementary Figures and Tables**

# Figure S1: Estimates of the percentage of male and female deaths combined attributed to obesity in the Netherlands, using world RRs, 1981–2013

# Figure S2: Age-standardised obesity prevalence by sex, 20–75+ yrs. in the Netherlands, 1981–2013

# Figure S3: BMI Mean values, 30–79 years, the Netherlands, 1981–2013

# Men

# Women

# Table S1: PAF estimates compared in men and women for the Netherlands 1981, 1991, 2001 and 2013, and 1981–2013

|  | 1981 | 1991 | 1993 | 2001 | 2013 |
| --- | --- | --- | --- | --- | --- |
| Men |  |  |  |  |  |
| Partially adjusted - world | 0.67% | 0.50% | 0.54% | 0.86% | 0.97% |
| Partially adjusted – Europe | 0.98% | 0.74% | 0.84% | 1.27% | 1.45% |
| Weighted sum method – world | 0.56% | 0.51% | 0.57% | 0.83% | 0.86% |
| Weighted sum method – Europe | 1.25% | 0.95% | 1.08% | 1.65% | 1.88% |
| Combined all-cause method – world | 1.00% | 0.75% | 0.81% | 1.27% | 1.43% |
| Adjusted CRA, recent – world | 0.66% | 0.75% | 0.82% | 1.16% | 1.29% |
| Adjusted CRA, less recent – world | 0.68% | 0.77% | 0.81% | 1.12% | 1.21% |
| Women |  |  |  |  |  |
| Partially adjusted - world | 0.50% | 0.59% | 0.51% | 0.85% | 0.94% |
| Partially adjusted – Europe | 0.59% | 0.89% | 0.77% | 1.27% | 1.37% |
| Weighted sum method – world | 0.60% | 0.62% | 0.58% | 0.95% | 0.98% |
| Weighted sum method – Europe | 0.92% | 1.08% | 0.94% | 1.56% | 1.68% |
| Combined all-cause method – world | 0.70% | 0.81% | 0.70% | 1.17% | 1.29% |
| Adjusted CRA, recent – world | 1.64% | 1.93% | 1.55% | 1.83% | 1.62% |
| Adjusted CRA, less recent – world | 1.64% | 1.74% | 1.47% | 1.71% | 1.48% |
